# Epicardial connections across the cavotricuspid isthmus

**DOI:** 10.1016/j.hrcr.2024.09.021

**Published:** 2024-10-04

**Authors:** Emily Janak, Alexander Turin, Weston Harkness, Alexander Green, Peter Santucci, Smit Vasaiwala

**Affiliations:** 1 Department of Cardiology, Loyola University Medical Center, Maywood, Illinois; 2 Department of Cardiology, University of Michigan, Ann Arbor, Michigan

**Keywords:** Atrial flutter, Ablation, Atrial mapping, Epicardial connections, CTI

**Key Teaching Points**

• Epicardial connections across the cavotricuspid isthmus (CTI) are important considerations in cases of difficult-to-achieve CTI block.
• With the increased availability and use of high-density mapping, identification of these circuits may be more feasible, and operators should consider epicardial connections across CTI lines in which first-pass block is not achieved.
• This precision may improve procedural efficiency and reduce complications by avoiding extensive perhaps unnecessary empiric ablations.

## Introduction

The cavotricuspid isthmus (CTI), is a critical connection for many right atrial (RA) macro-reentrant flutters and is a safe and effective ablation target. Bidirectional block across the CTI is the accepted endpoint of the procedure and may be difficult to achieve because of the presence of sub-Eustachian pouches, presence of a prominent Eustachian ridge, or enlarged pectinate muscles, among other challenges.^1^ Block can be confirmed with several approaches, such as differential pacing maneuvers or electroanatomic mapping.^2^^,^^3^ Epicardial connections involved in atrial arrhythmia have been described previously between the coronary sinus (CS) and left atrium (LA), into the carina of the pulmonary veins and Bachmann’s bundle in certain biatrial flutters.^4^, ^5^, ^6^ A single case series by Chaumont et al.^7^ describes epicardial connections in the RA across the CTI using intracardiac electrograms but without three-dimensional (3D) electroanatomic mapping (EAM).

We report the first case series of epicardial bridging across the CTI demonstrated with 3D EAM. All patients had persistent conduction across the CTI after linear ablation with epicardial “jump” of conduction demonstrated with EAM. Block across the CTI was successfully achieved by targeting these regions of endocardial breakout (earliest endocardial activation of conduction lateral or medial to the CTI) in all patients.

## Methods

Patients presented to the electrophysiology laboratory for catheter ablation of management of symptomatic atrial arrhythmias. All studies were performed while in a fasted state with appropriate anesthesia. Right and/or left femoral venous access was obtained with ultrasonographic guidance, and a decapolar catheter was placed into the CS (Biosense Webster, Irvine, CA). Trans-septal puncture was performed if necessary for concurrent left atrial arrhythmias. Three-dimensional electroanatomic mapping for all cases was performed using Carto (Biosense Webster, Irvine, CA), and intracardiac echocardiography (ICE) (Soundstar, Biosense Webster) was used to identify the CTI line and other anatomic structures. Atrial flutter (AFL) either was induced with programmed electrical stimulation or occurred spontaneously after ablation of other atrial arrhythmias. Typical AFL was verified with local activation time (LAT) mapping confirming counterclockwise activation around the tricuspid annulus. LAT mapping was performed using a high-density mapping catheter (Octaray, Biosense Webster, Irvine, CA) or point-by-point with a 4-mm-tip irrigated force-sensing catheter (SmartTouch, Biosense Webster) supported by a medium or large-curve Vizigo sheath (Biosense Webster). Lesions were delivered at 30–35 W for a target ablation index of 450–500. Block across the CTI was confirmed with differential pacing as described previously.^2^

When CTI block was not achieved as demonstrated by differential pacing from both sides of the line despite adequate linear ablation, LAT mapping was performed using an ablation or high-density mapping catheter. For patients in sinus rhythm, points were taken lateral to the CTI line during proximal CS pacing, while in counterclockwise AFL, data medial to the line were collected. In the event of identifying a gap on the line, the earliest signals identified on either side of the line were targeted for ablation; often these were found near prominent ridges or pouches or at either end of the CTI line. Epicardial connections were suspected when activation signals were noted 1–2 cm away from the line and emanated in all directions, including toward the CTI line. A low crista break can also be excluded by earliest focal activation lateral to the CTI line. Although high-density mapping provides detailed data and may be faster, we found that point-by-point mapping was equally effective in identification of epicardial CTI connections.

## Results

Five patients had evidence of typical counterclockwise AFL as verified with LAT mapping, and they are described here.

### Case 1

**Figure 1:**
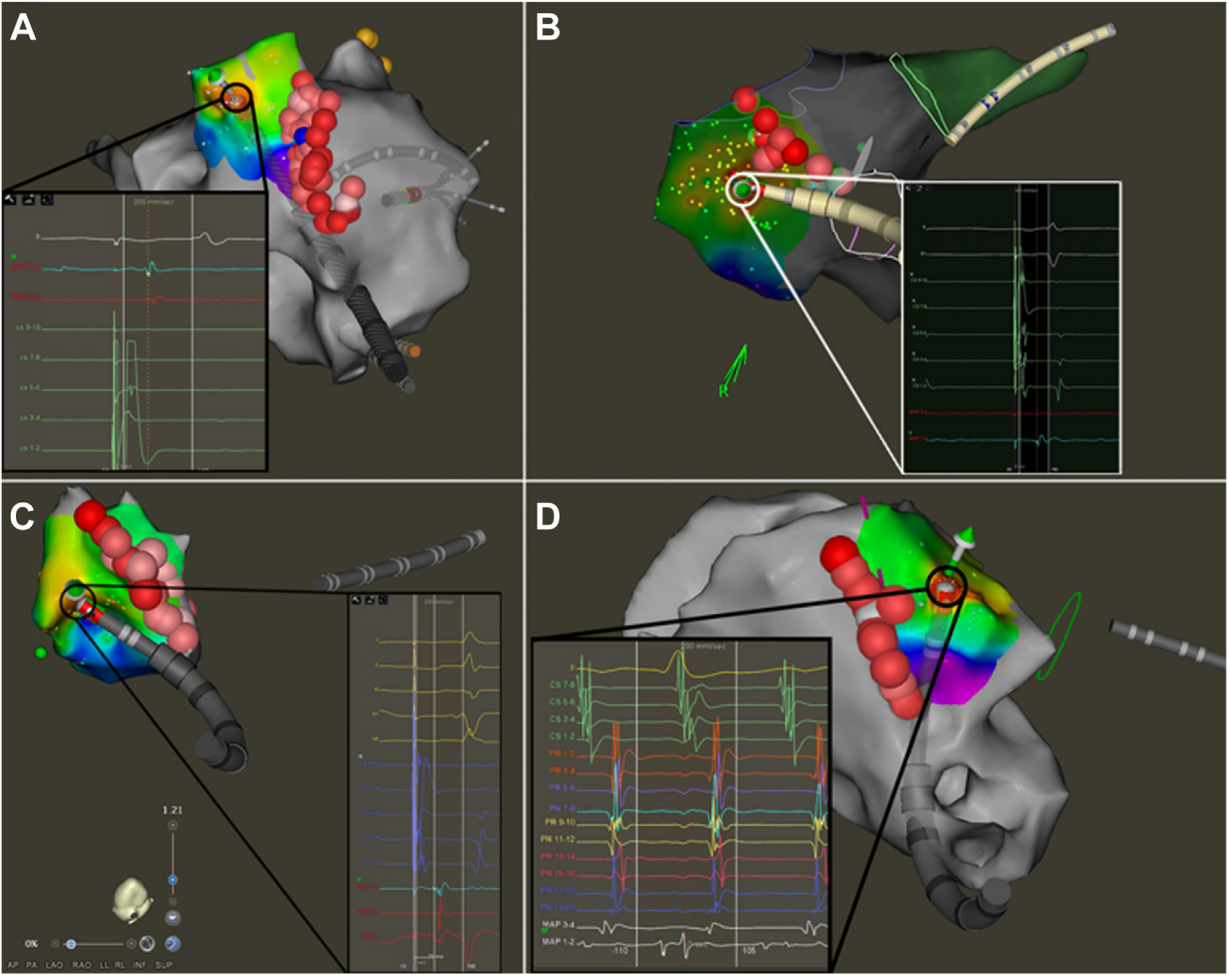
Local activation time mapping demonstrating early activation across the cavotricuspid isthmus (CTI) ablation line in the LAO caudal projection. (**A**), (**B**), and (**C**) correspond to case 1, 4, and 5, respectively, and they indicate epicardial connections lateral to the line as determined by proximal coronary sinus pacing as seen in the accompanying electrograms obtained by the ablation catheter at the site of earliest activation. **D:** Case 3 represents an epicardial connection seen during typical counterclockwise flutter with earliest activation medical to the CTI line.

A 63 year-old man with persistent AF presented for catheter ablation. After pulmonary vein isolation (PVI), atrial flutter was induced. LAT mapping demonstrated counterclockwise activation around the tricuspid annulus. Ablation was performed along the CTI at up to 35 W targeting an ablation index of 550, and the tachycardia terminated cleanly during ablation of the CTI. Despite result, block was not achieved across the CTI based on transisthmus time of 98 ms and differential pacing showing lateral right atrial time of 115 ms. LAT mapping lateral to the deployed ablation line during CS pacing showed early focal activation 1–2 cm lateral to the line (Figure 1A). Ablation at this region immediately extended the transisthmus time dramatically to 156 ms, and block was subsequently confirmed with differential pacing.

### Case 2

**Figure 2:**
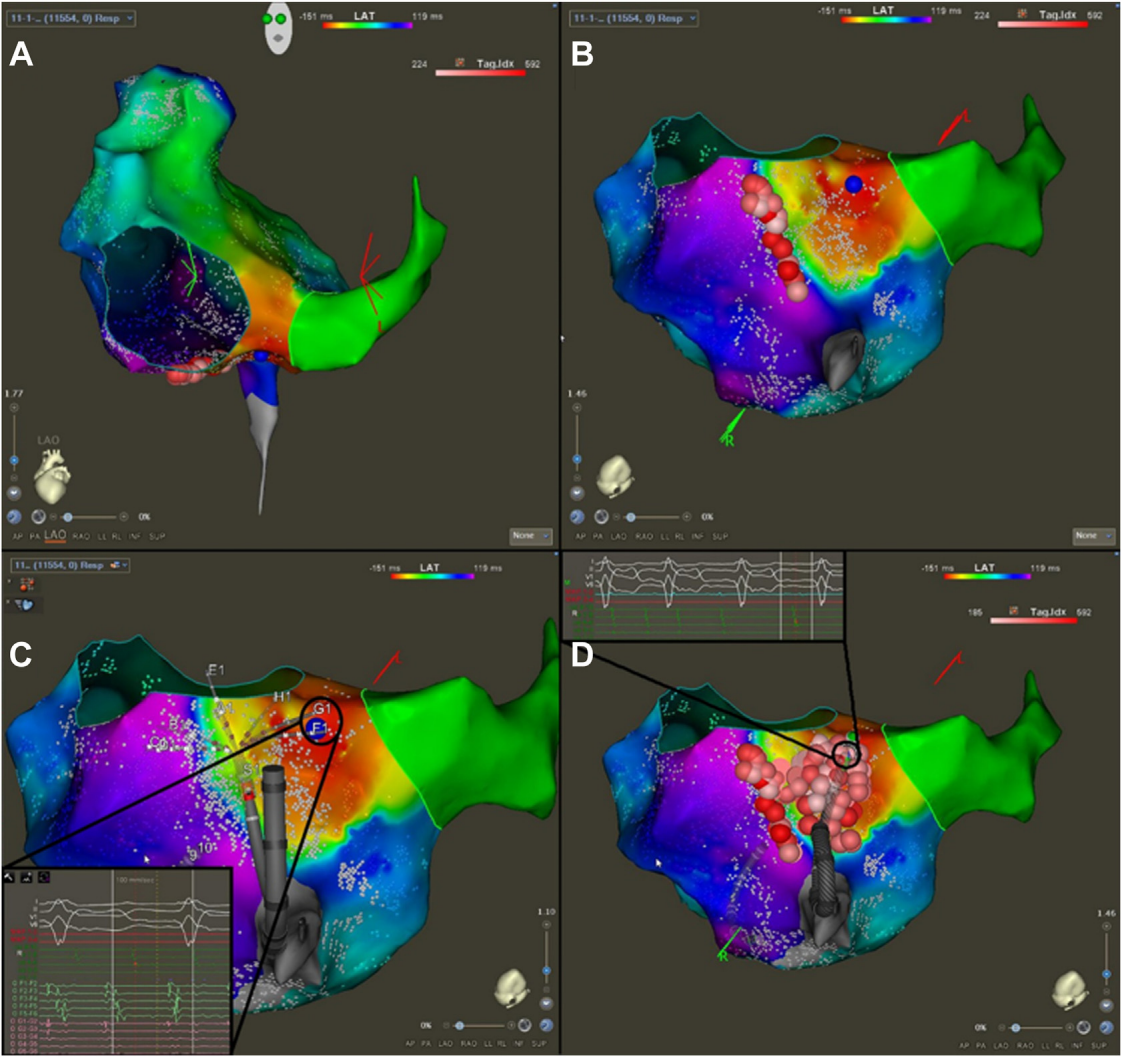
**A:** Local activation time map demonstrating counterclockwise typical flutter in the left anterior oblique (LAO) projection. **B:** LAO caudal projection showing early activation medial to the cavotricuspid isthmus line, suggestive of epicardial connection with accompanying electrogram seen in (**C**). **D:** Final lesion set including lesion and electrogram showing termination of atrial flutter in the area of medial epicardial connection. Extensive regional ablation was required to achieve block, suggestive of broad endocardial connections.

An 81 year-old man with persistent AF and AFL with multiple prior ablations presented for repeated catheter ablation for recurrent AT/AFL. He presented in sinus rhythm but with isoproterenol, AT was induced. LAT mapping was consistent with typical AFL, and ablation of the CTI was performed at up to 35 W for target ablation index of 550. Tachycardia persisted, and repeated LAT mapping demonstrated counterclockwise activation around the tricuspid annulus (Figure 2A and 2B) with early activation 1–2 cm medial to the CTI line, suggestive of epicardial jump (Figure 2C). Ablation at the site of the epicardial breakthrough resulted in termination of this atrial tachycardia (AT) to sinus rhythm, and the area was consolidated with further ablation (Figure 2D). Block across the CTI line was confirmed with LAT mapping and differential pacing.

### Case 3

A 66-year-old man with persistent atrial fibrillation (AF) and prior PVI ablation with recurrent AT/AF presented for AFL ablation. Activation in the CS was eccentric and LAT mapping of the LA demonstrated a focal AT emanating from the lateral mitral isthmus. This converted during mapping to an AFL that was now concentric in the CS. LAT mapping demonstrated typical counterclockwise AFL. Ablation on the CTI was performed with no effect on the tachycardia. Mapping of the medial exit was performed (Figure 1D), which showed a focal area of activation 1–2 cm medial to the line, and ablation at this point cleanly terminated tachycardia. Additional ablation lesions were performed along the line, and CTI block was confirmed with differential pacing. The previous LA focal AT was reinduced and successfully ablated at the lateral mitral annulus.

### Case 4

A 62-year-old man with a history of typical AFL with prior cardioversion, dilated cardiomyopathy with ejection fraction of 30% (presumed tachycardia-mediated), hypertension, obstructive sleep apnea, and morbid obesity presented for AFL ablation. The patient was not tolerating anesthesia well because of airway obstruction and agitation; therefore, given the typical 12-lead electrocardiogram morphology, empiric CTI ablation was performed without arrhythmia induction to expedite procedure time. Baseline transisthmus time was 72 ms. After linear ablation of the CTI, transisthmus time extended to 120 ms, and LAT mapping during proximal CS pacing demonstrated an area of early activation 1.5 cm lateral to the CTI line, suggestive of epicardial breakthrough (Figure 1B). Ablation was performed on this site with rapid extension of the transisthmus time to 153 ms. Block across the CTI was verified with differential pacing.

### Case 5

A 67-year-old man with a history of typical AFL with prior cardioversion owing to tachycardia-mediated cardiomyopathy (ejection fraction 35%) presented for AFL ablation. AFL was not inducible during the procedure. Given the typical appearance on a 12-lead electrocardiogram, ablation of the CTI was performed. Baseline transisthmus time with proximal CS pacing was 55 ms. After linear ablation, transisthmus time was 112 ms, suggesting incomplete block across the CTI. LAT mapping of the medial exit demonstrated a focal area of activation 1–2 cm lateral to the CTI line (Figure 1C). Ablation was performed in this area during CS pacing, which extended the transisthmus time dramatically to 180 ms. Block across the CTI was then confirmed with differential pacing.

## Discussion

Using 3D EAM and LAT mapping, these cases illustrate the presence of epicardial connections across an ablated CTI line that may pose challenges to obtain a bidirectional block. Often AFL will terminate with CTI ablation but without CTI block, as was the case in three of the cases presented. These showed early activation 1–2 cm lateral to the ablated CTI line during proximal CS pacing. Cases 2 and 3 demonstrated the reverse with early activation 1–2 cm medial to the CTI line during counterclockwise typical AFL, suggesting these connections may be bidirectional as well. In all cases, the site of connection was approximately 1–2 cm away from the ablation line which illustrates a distinct site of early activation rather than a breakout from an ablation gap in the line.

There has been minimal literature exploring epicardial connections across the CTI. Kobayashi et al.^8^ described as case of typical AFL refractory to initial CTI ablation. LAT mapping demonstrated early activation across the CTI medial to the line while in AFL, similar to cases 2 and 3. Higuchi et al.^9^ reported a similar finding in a patient presenting for his third redo-ablation for typical AFL, again noting early activation medial to the previous CTI that terminated tachycardia with ablation. Chaumont et al.^7^ published a five-patient case series in 2020 exploring the incidence of epicardial connections between low right atrium and remote right atrial region or coronary sinus musculature. Using multipolar catheters and evaluation of electrograms rather than LAT mapping, the authors eloquently demonstrated the presence of epicardial connections across the CTI.

Epicardial connections between the CS and LA have been described previously,^4^ and it is important to consider the presence of epicardial bridging in situations in which block across the CTI is challenging to attain. Incidence of epicardial CTI connections has not been well defined, but it is estimated at approximately 0.2%–0.3%.^7^ As it is common to extend ablation medially or laterally when block is not achieved, it is possible that these connections are ablated incidentally and the true incidence is even higher. With the availability and routine use of high-density mapping, it may also be easier to identify epicardial connections that were previously overlooked.

## Conclusion

Epicardial connections across the CTI are important considerations in cases of difficult-to-achieve CTI block. There have been few reports highlighting this finding, and this is the first case series to illustrate epicardial breakthrough across the CTI using high-density LAT mapping. With the increased availability and use of high-density mapping, identification of these circuits may be more feasible, and operators should consider epicardial connections across CTI lines in which first-pass block is not achieved. This precision may improve procedural efficiency and may reduce complications by avoiding extensive perhaps unnecessary empiric ablations.

## Disclosures

The authors have no conflicts to disclose.
